# The Diverse Role of NK Cells in Immunity to *Toxoplasma gondii* Infection

**DOI:** 10.1371/journal.ppat.1005396

**Published:** 2016-02-25

**Authors:** Jason P. Gigley

**Affiliations:** Department of Molecular Biology, University of Wyoming, Laramie, Wyoming, United States of America; University of Wisconsin Medical School, UNITED STATES

## Introduction

The obligate intracellular parasite *Toxoplasma gondii* (*T*. *gondii*), found in ~30% of humans worldwide, is a significant health risk for people with HIV/AIDS, people undergoing chemotherapy treatment or organ transplantation, and for developing fetuses as a result of congenital infection [1]. Infection can cause death, blindness, spontaneous abortion, or mental retardation and is correlated with behavior and neurocognitive changes [2]. No therapies exist to prevent or clear parasite infection, which is lifelong. CD8 T cell interferon (IFN)γ is the major mechanism of protection [3,4]; however, many immune factors contribute to successful parasite control. One is the natural killer cell (NK cell) [5]. NK cells are considered group 1 innate lymphoid cells (ILCs) and provide defense against tumors and intracellular pathogens (viruses, bacteria, and parasites) [6]. They use surface receptors (activating, inhibitory, and cytokine) to survey host cells and tissues for damage or infection. Receptor engagement stimulates killing of diseased target cells (cytotoxicity) and initiating IFNγ production. Activating receptors recognize specific ligands expressed on target cell surfaces and activate via cytoplasmic immunoreceptor tyrosine-based activation motifs (ITAM) or associated adapter molecules DAP10 and DAP12 [7]. Inhibitory receptors inhibit by assessing self through MHC Class I (MHCI) recognition and, when triggered, recruit ITAM-antagonizing phosphatases SHIP1 and SHP1 via cytoplasmic immunoreceptor tyrosine-based inhibitory motifs (ITIM) [8]. Inflammatory cytokines IFNα/β, Interleukin (IL)-2, IL-12, IL-15, and IL-18 synergize with activating receptor signals or stimulate NK cell activation alone [7]. Other functional surface proteins include FcγRs, Fas, and tumor necrosis factor-related apoptosis-inducing ligand (TRAIL). Ultimately, these surface proteins regulate NK cell function depending upon the stimulatory environment. Studies of NK cell responses to *T*. *gondii* have broadly impacted parasite immunology and NK cell fields. Thus, *T*. *gondii* is an excellent and relevant model to investigate NK cell biology. This model will be important in future studies, given newly emerging NK cell adaptive immune and regulatory roles and their therapeutic potential.

## Protective Role of NK Cells in Primary *T*. *gondii* Infection

**Fig 1:**
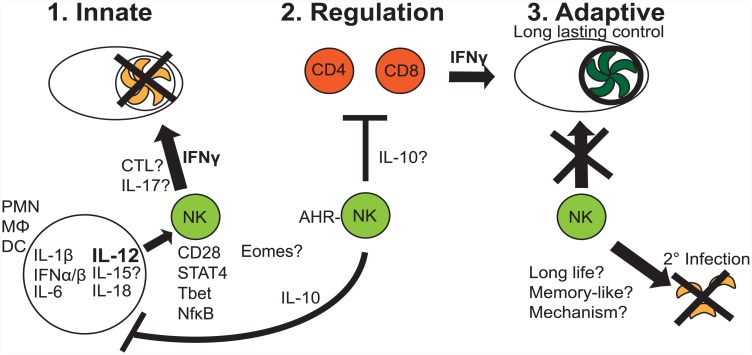
Multiple roles for NK cells during *T*. *gondii* infection. Natural killer (NK) cells function in different phases of immunity in response to parasite infection. **Step 1: Innate**. During the innate response, *T*. *gondii* infection stimulated production of inflammatory cytokines IL-1β, IFNα/β, IL-6, IL-12, IL-15, and IL-18, driving NK cell production of IFNγ. This results in early control of parasite infection by targeting intracellular parasites. IL-6 can stimulate NK cell IL-17 production. The importance of NK cell IL-17 is not well understood. Cytotoxic (CTL) response by NK cells is also induced; however, the importance of this function for control of acute parasite infection is not well known. Other factors important for NK cell responses include CD28, STAT4, Tbet, and NfκB family members (cRel, p50). Eomesodermin (Eomes) role is unclear. **Step 2: Regulation**. NK cells produce IL-10 and regulate innate responses by down-regulating IL-12 and possibly other cytokines. This is aryl hydrocarbon receptor (AHR)-dependent. Whether NK cell IL-10 can impact CD4 and CD8 T cell responses is not known. **Step 3: Adaptive**. NK cells can participate in adaptive immunity as memory-like cells. NK cells may be important for (2°) secondary *T*. *gondii* infections. Whether NK cells that experience *T*. *gondii* infection early live long-term or develop memory-like features and the mechanisms behind these cell-intrinsic fates are unknown.

Early *T*. *gondii* studies added to the understanding of NK cells in immunity because they showed that a parasite, in addition to tumor cells and viral infection, could stimulate NK cell activity. NK cells were originally identified as naturally cytotoxic cells that kill tumor cells [9]. Acute and chronic *T*. *gondii* infection stimulates NK cell cytotoxicity regardless of mouse strain [10,11]. *T*. *gondii* soluble and particulate component injections stimulate cytotoxic NK cell responses in mouse and human cells [12,13]. Using NK cell-depleting antibodies, NK cells were shown to be essential for early parasite control [5]. Just prior, NK cells were shown to produce IFNγ, but it was unclear how IFNγ contributed to the immune response. Subsequent studies showed that NK cell IFNγ was a dominant, early protective mechanism (Fig 1, Step 1) [5,14].

Although *T*. *gondii* infection stimulates NK cell cytotoxicity, its importance for control is unclear [10]. Perforin-deficient mice, which globally lack cytotoxicity, survive avirulent parasite infection, likely because of intact IFNγ production [15]. However, long-term survival is impaired. Parasite-induced NK cell responses cross-protect against H5N1 influenza infection and established B16F10 melanoma [16,17]. Thus, the parasite does induce effective cytotoxic NK cells. Currently, testing NK cell cytotoxicity for parasite control is difficult because of a lack of experimental tools. NK cells in *T*. *gondii* infection produce IL-17. *T*. *gondii* NK cell immunity may involve IL-17 production, stimulated by IL-6 (Fig 1, Step 1) [18]. Whether IL-17 is protective or contributes to immune pathology is unknown.

NK cell help to T cells was not realized until a *T*. *gondii* study demonstrated they helped CD8 T cells in absence of CD4 T cells [19]. NK cell–dendritic cell (DC) interactions are known to stimulate development of dendritic cell type 1 (DC1). *T*. *gondii*-stimulated NK cell IFNγ drives inflammatory DC differentiation in initial parasite infection to boost DC activation of T cells [20]. Thus, NK cell IFNγ directly controls *T*. *gondii* and augments T cell responses.

## Mechanisms of NK Cell Activation during *T*. *gondii* Infection

*T*. *gondii* studies have been instrumental for understanding NK cell activation mechanisms. One mechanism is IL-12 induction of IFNγ and the importance of this axis for NK cell function [21]. *T*. *gondii*-controlling NK cell IFNγ is IL-12–dependent (Fig 1, Step 1). *T*. *gondii* studies identified additional factors important for NK cell activation. These include cytokines IFNα/β, IL-1β, IL-2, IL-7, IL-18, and tumor necrosis factor (TNF)-α [14,21–24], which synergize with or substitute for IL-12. IL-1β is required for IL-12–induced NK cell IFNγ, and IL-2 and IL-18 overcome IL-12–dependent NK cell activation in STAT4-deficient mice [23,25]. IL-15 is important for NK cell development, peripheral maintenance, and function. However, *T*. *gondii* infection was the first model to show intact NK cell IFNγ in IL-15–deficient mice (Fig 1, Step 1) [26]. Costimulatory molecules and transcription factors also impact *T*. *gondii*-induced NK cell responses. CD28 on NK cells synergizes with signals from IL-15 [27]. NFκB family members c-Rel and p50 can regulate NK cell proliferation and IFNγ production [28]. T-box transcription factor T-bet, paramount for T cell IFNγ production, is not required for parasite-induced local NK cell IFNγ [29]. Whether Eomes is important and how T-bet and Eomes interplay works will be interesting to address.

**Fig 2:**
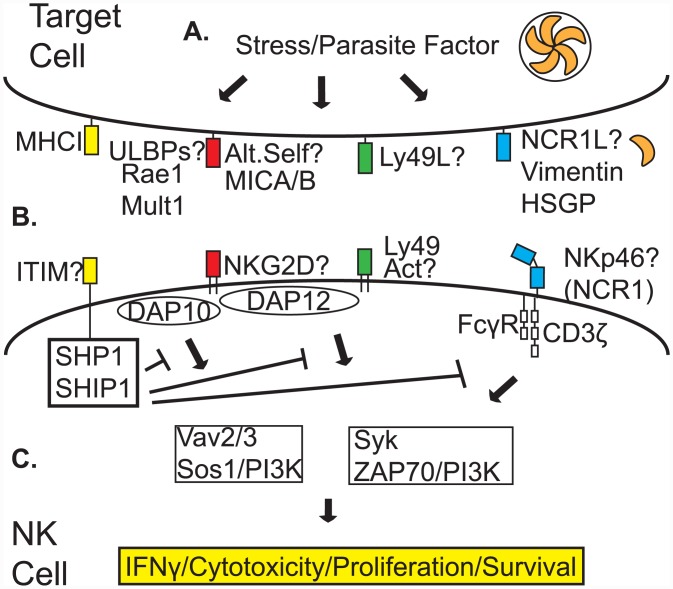
Possible activating receptor and NK cell subpopulation involvement in recognition of *T*. *gondii*-infected cells. The subpopulation of NK cells important for IFNγ-dependent protection, defined by specific activating (immunoreceptor tyrosine-based activating motif [ITAM]) receptors, is unknown. **A.** Infection of a target cell by *T*. *gondii* could induce stress, resulting in expression of ULBPs (Rae1, Mult1, or others), or alter self (MICA/B), Ly49-specific ligands, and/or NCR1 ligands (NCR1L, possible molecules vimentin or heparan sulfate glycoproteins [HSGP]). **B.** ULBPs or altered self-molecules would be recognized by NKG2D. Parasite-produced Ly49 ligands would be recognized by Ly49H or Ly49D. Host-derived NCR1 ligands would be recognized by NKp46 (NCR1). MHC Class I (MHCI) could be recognized by immunoreceptor tyrosine-based inhibitory motif (ITIM) receptors and SHP1/SHIP1 could impact signaling. **C.** NK cell-activating ligands that are recognized would activate the NK cell to produce cytokines (IFNγ), be cytotoxic, proliferate, and promote survival via signaling from either NKG2D-associated DAP10 or DAP12-dependent activation of Vav2/3/Sos1/PI3K or Syk/ZAP70/PI3K-dependent pathways, respectively, Ly49-associated DAP12-dependent activation of Syk/ZAP70/PI3K or NKp46-associated FcγR, and CD3ζ chain-dependent activation of Syk/ZAP70/PI3K signaling. Additional receptors not shown in figure include CD94/NKG2C, 2B4, FcRγIII, TRAIL, and IL-12R.

A major question is whether dominant, protective, *T*. *gondii*-specific NK cell subpopulations exist. These subpopulations are defined by expressed surface receptors that tune responsiveness via differing signaling mechanisms (Fig 2C). Are there (1) specific NK cell receptors and signaling pathways required for activation; (2) parasite-derived ligands or infected, host stress-induced molecules required; and (3) specific protective NK cell populations? Published data suggests licensed NK cells (activating and inhibitory receptor-expressing cells) produce IFNγ during parasite infection (Fig 2) [30]. Other studies implicate NKG2D ligands Rae1 and Mult1 (Fig 2B) for activation [31]. Natural cytotoxicity triggering receptor (NCR)1 (NKp46) is required for nonconventional NK cell ILC1 IFNγ production against *T*. *gondii* in the gut (Fig 2B) [32]. This could also be true for NK cell IFNγ. Additional activating receptors in mice include Ly49, CD94/NKG2C, 2B4, FcγRIII, and TRAIL. In sum, mechanistic studies of NK cell activation with *T*. *gondii* have impacted the NK cell field. These include IL-12/IFNγ axis, IL-15–independent NK cell infection responses, costimulation, and T-bet role in NK cell dependent protection.

## Regulatory Role of NK Cells in Acute *T*. *gondii* Infection

NK cell immunoregulation has recently come to light [33]. Mechanisms are not defined, but they are likely important to prevent inflammation-dependent pathology. *T*. *gondii* studies have been important in understanding this process [33]. *T*. *gondii* induces robust inflammation that is driven by high innate cell (DC, macrophage, neutrophil (PMN)-produced IL-12 [1]. Unregulated inflammation results in immunopathology in murine parasite infection. IL-10 is important for counterbalancing this inflammatory response [34]. NK cells are a source of IL-10 in systemic *T*. *gondii* infection (Fig 1, Step 2). IL-10 is produced by IFNγ+ NK cells and is dependent upon IL-12 and the aryl hydrocarbon receptor [35]. Importantly, NK IL-10 feedback on DCs limits IL-12 production, thus regulating inflammation [34]. Long-term consequences of NK cell IL-10 are unknown and could impact quality and magnitude of adaptive immunity to this parasite (Fig 1, Step 2). Additional studies show that NK cell IFNγ in bone marrow impacts mucosal and systemic regulatory monocyte programming [36]. Thus, NK cells control parasites and regulate innate immunity to *T*. *gondii*.

## Emerging NK Cell Roles in Immunity and Relevance to *T*. *gondii*

The paradigm that NK cells are only innate immune cells is changing. Evidence supports their development of memory-like traits. This was first shown in contact hypersensitivity reactions following chemical hapten exposure in mice lacking T cells or B cells, then after in murine cytomegalovirus (MCMV) infection [37]. MCMV infection-induced memory-like NK cells are dependent upon IL-12. These memory-like features can also be developed after exposure to inflammatory cytokines IL-12, IL-15, and IL-18. Human studies have identified NKG2C+ NK cells to have memory-like traits [38]. Human NK cells can be stimulated by CMV and HIV infections as well as by cytokine cocktail stimulation [37]. In nonviral infections, evidence for NK cell memory-like characteristics is less clear. In *Plasmodium* and *Listeria* secondary challenges, memory T cell IFNγ is required for secondary NK cell responses [37]. Interestingly, β2m-deficient mice (CD8 T cell-deficient) develop NK cell-dependent protective immunity against *T*. *gondii* challenge after immunization with temperature-sensitive mutant ts-4 parasites [5]. This suggests NK cells participate in adaptive immune responses and may acquire adaptive immune features. However, whether early-responding NK cells differentiate into bona fide memory-like cells specific to *T*. *gondii* and mechanisms underlying differentiation are unknown (Fig 1, Step 3). We have preliminary evidence of NK cell-dependent protection against secondary *T*. *gondii* infection and adoptively transferred protection of NK cell-deficient (RAG2/cγchain-deficient) mice with *T*. *gondii*-experienced NK cells. Although much needs addressing, early evidence suggests *T*. *gondii*-induced NK cells may develop features of adaptive immune cells.

## Conclusions

Importance of NK cell immunity against pathogens is expanding from acute control to regulation and now adaptive memory-like responses. *T*. *gondii* represents a unique pathogen model to better understand this cell type in immunity. NK cells are required for acute *T*. *gondii* control, regulate inflammation via IL-10, and may contribute to adaptive immune responses. Thus, NK cells during *T*. *gondii* infection have multiple and complex roles at all phases of immunity to this parasite.

## References

[1] Hunter CA, Sibley LD (2012) Modulation of innate immunity by Toxoplasma gondii virulence effectors. Nat Rev Microbiol 10: 766–778. 10.1038/nrmicro2858 23070557 PMC3689224

[2] Dickerson F, Stallings C, Origoni A, Katsafanas E, Schweinfurth L, et al (2014) Antibodies to Toxoplasma gondii and cognitive functioning in schizophrenia, bipolar disorder, and nonpsychiatric controls. J Nerv Ment Dis 202: 589–593. 10.1097/NMD.0000000000000166 25010110

[3] Suzuki Y, Orellana MA, Schreiber RD, Remington JS (1988) Interferon-gamma: the major mediator of resistance against Toxoplasma gondii. Science 240: 516–518. 3128869 10.1126/science.3128869

[4] Suzuki Y, Remington JS (1988) Dual regulation of resistance against Toxoplasma gondii infection by Lyt-2+ and Lyt-1+, L3T4+ T cells in mice. J Immunol 140: 3943–3946. 3259601

[5] Denkers EY, Gazzinelli RT, Martin D, Sher A (1993) Emergence of NK1.1+ cells as effectors of IFN-gamma dependent immunity to Toxoplasma gondii in MHC class I-deficient mice. J Exp Med 178: 1465–1472. 8228800 10.1084/jem.178.5.1465 PMC2191244

[6] Spits H, Artis D, Colonna M, Diefenbach A, Di Santo JP, et al (2013) Innate lymphoid cells—a proposal for uniform nomenclature. Nat Rev Immunol 13: 145–149. 10.1038/nri3365 23348417

[7] Lanier LL (2005) NK cell recognition. Annu Rev Immunol 23: 225–274. 15771571 10.1146/annurev.immunol.23.021704.115526

[8] Daeron M, Jaeger S, Du Pasquier L, Vivier E (2008) Immunoreceptor tyrosine-based inhibition motifs: a quest in the past and future. Immunol Rev 224: 11–43. 10.1111/j.1600-065X.2008.00666.x 18759918

[9] Herberman RB, Nunn ME, Lavrin DH (1975) Natural cytotoxic reactivity of mouse lymphoid cells against syngeneic acid allogeneic tumors. I. Distribution of reactivity and specificity. Int J Cancer 16: 216–229. 50294 10.1002/ijc.2910160204

[10] Kamiyama T, Hagiwara T (1982) Augmented followed by suppressed levels of natural cell-mediated cytotoxicity in mice infected with Toxoplasma gondii. Infect Immun 36: 628–636. 6177634 10.1128/iai.36.2.628-636.1982 PMC351275

[11] Hauser WE Jr., Sharma SD, Remington JS (1982) Natural killer cells induced by acute and chronic toxoplasma infection. Cell Immunol 69: 330–346. 6980721 10.1016/0008-8749(82)90076-4

[12] Hauser WE Jr., Sharma SD, Remington JS (1983) Augmentation of NK cell activity by soluble and particulate fractions of Toxoplasma gondii. J Immunol 131: 458–463. 6190921

[13] Sharma SD, Verhoef J, Remington JS (1984) Enhancement of human natural killer cell activity by subcellular components of Toxoplasma gondii. Cell Immunol 86: 317–326. 6587935 10.1016/0008-8749(84)90386-1

[14] Sher A, Oswald IP, Hieny S, Gazzinelli RT (1993) Toxoplasma gondii induces a T-independent IFN-gamma response in natural killer cells that requires both adherent accessory cells and tumor necrosis factor-alpha. J Immunol 150: 3982–3989. 8473745

[15] Denkers EY, Yap G, Scharton-Kersten T, Charest H, Butcher BA, et al (1997) Perforin-mediated cytolysis plays a limited role in host resistance to Toxoplasma gondii. J Immunol 159: 1903–1908. 9257855

[16] Baird JR, Byrne KT, Lizotte PH, Toraya-Brown S, Scarlett UK, et al (2013) Immune-mediated regression of established B16F10 melanoma by intratumoral injection of attenuated Toxoplasma gondii protects against rechallenge. J Immunol 190: 469–478. 10.4049/jimmunol.1201209 23225891 PMC3529845

[17] O'Brien KB, Schultz-Cherry S, Knoll LJ (2011) Parasite-mediated upregulation of NK cell-derived gamma interferon protects against severe highly pathogenic H5N1 influenza virus infection. J Virol 85: 8680–8688. 10.1128/JVI.05142-11 21734055 PMC3165849

[18] Passos ST, Silver JS, O'Hara AC, Sehy D, Stumhofer JS, et al (2010) IL-6 promotes NK cell production of IL-17 during toxoplasmosis. J Immunol 184: 1776–1783. 10.4049/jimmunol.0901843 20083665 PMC3757499

[19] Combe CL, Curiel TJ, Moretto MM, Khan IA (2005) NK cells help to induce CD8(+)-T-cell immunity against Toxoplasma gondii in the absence of CD4(+) T cells. Infect Immun 73: 4913–4921. 16041005 10.1128/IAI.73.8.4913-4921.2005 PMC1201207

[20] Goldszmid RS, Caspar P, Rivollier A, White S, Dzutsev A, et al (2012) NK cell-derived interferon-gamma orchestrates cellular dynamics and the differentiation of monocytes into dendritic cells at the site of infection. Immunity 36: 1047–1059. 10.1016/j.immuni.2012.03.026 22749354 PMC3412151

[21] Gazzinelli RT, Hieny S, Wynn TA, Wolf S, Sher A (1993) Interleukin 12 is required for the T-lymphocyte-independent induction of interferon gamma by an intracellular parasite and induces resistance in T-cell-deficient hosts. Proc Natl Acad Sci U S A 90: 6115–6119. 8100999 10.1073/pnas.90.13.6115 PMC46878

[22] Hunter CA, Subauste CS, Van Cleave VH, Remington JS (1994) Production of gamma interferon by natural killer cells from Toxoplasma gondii-infected SCID mice: regulation by interleukin-10, interleukin-12, and tumor necrosis factor alpha. Infect Immun 62: 2818–2824. 7911785 10.1128/iai.62.7.2818-2824.1994 PMC302887

[23] Hunter CA, Chizzonite R, Remington JS (1995) IL-1 beta is required for IL-12 to induce production of IFN-gamma by NK cells. A role for IL-1 beta in the T cell-independent mechanism of resistance against intracellular pathogens. J Immunol 155: 4347–4354. 7594594

[24] Hunter CA, Gabriel KE, Radzanowski T, Neyer LE, Remington JS (1997) Type I interferons enhance production of IFN-gamma by NK cells. Immunol Lett 59: 1–5. 9334850 10.1016/s0165-2478(97)00091-6

[25] Cai G, Radzanowski T, Villegas EN, Kastelein R, Hunter CA (2000) Identification of STAT4-dependent and independent mechanisms of resistance to Toxoplasma gondii. J Immunol 165: 2619–2627. 10946290 10.4049/jimmunol.165.5.2619

[26] Lieberman LA, Villegas EN, Hunter CA (2004) Interleukin-15-deficient mice develop protective immunity to Toxoplasma gondii. Infect Immun 72: 6729–6732. 15501812 10.1128/IAI.72.11.6729-6732.2004 PMC523054

[27] Hunter CA, Ellis-Neyer L, Gabriel KE, Kennedy MK, Grabstein KH, et al (1997) The role of the CD28/B7 interaction in the regulation of NK cell responses during infection with Toxoplasma gondii. J Immunol 158: 2285–2293. 9036976

[28] Tato CM, Mason N, Artis D, Shapira S, Caamano JC, et al (2006) Opposing roles of NF-kappaB family members in the regulation of NK cell proliferation and production of IFN-gamma. Int Immunol 18: 505–513. 16481345 10.1093/intimm/dxh391 PMC1800429

[29] Harms Pritchard G, Hall AO, Christian DA, Wagage S, Fang Q, et al (2015) Diverse roles for T-bet in the effector responses required for resistance to infection. J Immunol 194: 1131–1140. 10.4049/jimmunol.1401617 25556247 PMC4297724

[30] Goldszmid RS, Bafica A, Jankovic D, Feng CG, Caspar P, et al (2007) TAP-1 indirectly regulates CD4+ T cell priming in Toxoplasma gondii infection by controlling NK cell IFN-gamma production. J Exp Med 204: 2591–2602. 17923502 10.1084/jem.20070634 PMC2118487

[31] Guan H, Moretto M, Bzik DJ, Gigley J, Khan IA (2007) NK cells enhance dendritic cell response against parasite antigens via NKG2D pathway. J Immunol 179: 590–596. 17579080 10.4049/jimmunol.179.1.590

[32] Klose CS, Flach M, Mohle L, Rogell L, Hoyler T, et al (2014) Differentiation of type 1 ILCs from a common progenitor to all helper-like innate lymphoid cell lineages. Cell 157: 340–356. 10.1016/j.cell.2014.03.030 24725403

[33] Vivier E, Ugolini S (2009) Regulatory natural killer cells: new players in the IL-10 anti-inflammatory response. Cell Host Microbe 6: 493–495. 10.1016/j.chom.2009.12.001 20006835

[34] Perona-Wright G, Mohrs K, Szaba FM, Kummer LW, Madan R, et al (2009) Systemic but not local infections elicit immunosuppressive IL-10 production by natural killer cells. Cell Host Microbe 6: 503–512. 10.1016/j.chom.2009.11.003 20006839 PMC2796259

[35] Wagage S, John B, Krock BL, Hall AO, Randall LM, et al (2014) The aryl hydrocarbon receptor promotes IL-10 production by NK cells. J Immunol 192: 1661–1670. 10.4049/jimmunol.1300497 24403534 PMC3955958

[36] Askenase MH, Han SJ, Byrd AL, Morais da Fonseca D, Bouladoux N, et al (2015) Bone-Marrow-Resident NK Cells Prime Monocytes for Regulatory Function during Infection. Immunity 42: 1130–1142. 10.1016/j.immuni.2015.05.011 26070484 PMC4472558

[37] Sun JC, Ugolini S, Vivier E (2014) Immunological memory within the innate immune system. EMBO J 33: 1295–1303. 10.1002/embj.201387651 24674969 PMC4194120

[38] Foley B, Cooley S, Verneris MR, Curtsinger J, Luo X, et al (2012) Human cytomegalovirus (CMV)-induced memory-like NKG2C(+) NK cells are transplantable and expand in vivo in response to recipient CMV antigen. J Immunol 189: 5082–5088. 10.4049/jimmunol.1201964 23077239 PMC3490031

